# Invisible excess of sense in social interaction

**DOI:** 10.3389/fpsyg.2014.01081

**Published:** 2014-09-30

**Authors:** Alice Koubová

**Affiliations:** Department of Contemporary Continental Philosophy, Institute of Philosophy of the Academy of Sciences of the Czech RepublicPrague, Czech Republic

**Keywords:** participatory sense-making, enactive theory, Merleau-Ponty, invisibility, opacity, (Inter)acting with the inner partner, performativity, dramaturgical analysis

## Abstract

The question of visibility and invisibility in social understanding is examined here. First, the phenomenological account of expressive phenomena and key ideas of the participatory sense-making theory are presented with regard to the issue of visibility. These accounts plead for the principal visibility of agents in interaction. Although participatory sense-making does not completely rule out the existence of opacity and invisible aspects of agents in interaction, it assumes the capacity of agents to integrate disruptions, opacity and misunderstandings in mutual modulation. Invisibility is classified as the dialectical counterpart of visibility, i.e., as a lack of sense whereby the dynamics of perpetual asking, of coping with each other and of improvements in interpretation are brought into play. By means of empirical exemplification this article aims at demonstrating aspects of invisibility in social interaction which complement the enactive interpretation. Without falling back into Cartesianism, it shows through dramaturgical analysis of a practice called “(Inter)acting with the inner partner” that social interaction includes elements of opacity and invisibility whose role is performative. This means that opacity is neither an obstacle to be overcome with more precise understanding nor a lack of meaning, but rather an excess of sense, a “hiddenness” of something real that has an “active power” (Merleau-Ponty). In this way it contributes to on-going social understanding as a hidden potentiality that naturally enriches, amplifies and in part constitutes human participation in social interactions. It is also shown here that this invisible excess of sense already functions on the level of self-relationship due to the essential self-opacity and self-alterity of each agent of social interaction. The analysis consequently raises two issues: the question of the enactive ethical stance toward the alterity of the other and the question of the autonomy of the self-opaque agent.

## INTRODUCTION

Starting from a basic agreement with key ideas of enactive theory of social understanding, especially with the concept of participatory sense-making, this paper presents a detailed examination of the question of visibility and invisibility of agents in social interaction. In order to avoid Cartesian homuncularity, enactive theory pleads for the principal visibility of agents, the *a priori* given tendency to understand each other, and their capacity to integrate disruptions, opacity and misunderstandings in mutual modulation. While it does not fully deny opacity and invisible aspects of agents in interaction, it regards these as the dialectical counterpart of visibility, as a lack of sense that brings about the dynamics of social understanding in the form of asking, of coping with each other, and of improving interpretations. The aim of this article is to focus on an aspect of invisibility in social interaction that complements the enactive interpretation. Without lapsing into Cartesianism, I wish to exemplify several levels of invisibility in social interaction (physical, social, self-relational, and intersubjective). In particular, I wish to analyze a hypothesis according to which there is an aspect of invisibility that functions as a subtle *source* of ungraspable meaning, as an excess of sense, whose function is *performative*. This aspect, I argue, contributes to on-going social understanding as a hidden potentiality that naturally enriches, amplifies and in part constitutes human participation in social interactions. As the second part of the research I wish to draw upon experimental work to make it clear that an agent’s invisibility (opacity) is present already in the self-relationship and grounds the non-trivial structure of self-alterity.

I wish to demonstrate these aspects of social understanding on the basis of qualitative research accomplished within longitudinal studies of theatricality, performativity, and art-based practices at the Institute for Research and Study of Authorial Acting (IRSAA), at the Academy of Performing Arts in Prague and the Academy of Sciences of the Czech Republic. The research limits itself only to one experiment called “(Inter)acting with the inner partner” and its potential to exemplify self-alterity and the invisible excess of sense. Methodologically I mainly draw upon the research of the sociologist Goffman (1956) and his dramaturgical analysis (see also Hare and Blumberg, 1988), but important tools for the present study are also the examination of symbolic interaction in everyday life, the phenomenological method and participatory observation.

## THEORETICAL BACKGROUND

The problem of social cognition has been one of the most burning issues in psychology and cognitive science over the last several decades. The original “problem of other minds” that presupposes the existence of hidden mental, interior and private space represented secondarily through different linguistic, corporeal, and gestural manifestations has been widely criticized. The critique arose in phenomenological philosophy and has undergone further elaboration and development in the theory of embodied and enactive cognition.

As regards the phenomenological response to the problem of other minds, already in Scheler (1973 [1912], 232–234) proposed the concept of expressive unity (*Ausdruckseinheit*). He attempted to show that expression (corporeal behavior, action, gestures, discursive articulation) is not only a secondary visible manifestation of a *psyche*, but at least an integral part thereof. This statement is further developed by many other authors. Plessner (2003 [1941], 261) says that the unity of an expressive phenomenon is given by “indifference between content and form,” as is evident in the case of primary intersubjective expressive phenomena, such as laughter or crying, where the sign and meaning cannot be linked together arbitrarily. We may refer as well to Maurice Merleau-Ponty who assumes: “We must reject the prejudice which makes ‘inner realities’ out of love, hate or anger, leaving them accessible to one single witness: the person who feels them. Anger, shame, hate, and love are not psychic facts hidden at the bottom of another’s consciousness: they are types of behavior or styles of conduct which are visible from the outside. They exist on this face or in those gestures, not hidden behind them” (Merleau-Ponty, 1964a, 52–53). In this way phenomenology assumes that social cognition has to do with perception (*aisthesis*) and that a human being is in principle “visible” to other human beings.

The enactive theory of embodied cognition moves in a similar direction. It attempts to overcome Cartesianism and its third-person paradigm of social cognition understood as a passive observation of others’ behavior. As many have shown (Varela et al., 1991; Thompson and Varela, 2001; Gallagher and Varela, 2003; Thompson, 2007; Hutto, 2013; Hutto and Myin, 2013) the mind cannot be reduced to brain processes or internal representations, but is “an ongoing and situated activity” (De Jaegher and Di Paolo, 2007, 486). This entails that social understanding should be understood as “an interactional and intercorporeal process in which both partners are immersed and in which the process of interacting itself plays a leading role for the understanding… In short: social cognition emerges from embodied social interaction or, in Merleau-Ponty’s term, from intercorporeality” (Fuchs and De Jaegher, 2009, 469).

This familiar context is important for the purpose of this paper in one particular respect, namely as regards the principal visibility of other minds. The enactive theory opposes the Theory Theory (Premack and Woodruff, 1978; Baron-Cohen et al., 1986; Antonietti et al., 2006) and the Simulation Theory (Gordon, 1996; Dokic and Proust, 2002; Goldman, 2006) that share the common presupposition of “homuncularity, the absence of body” (De Jaegher and Di Paolo, 2007, 485), in other words the idea that “[human beings] are hidden from each other in principle” (Fuchs and De Jaegher, 2009, 467). Enactivism explains this presupposition of the Theory Theory and the Simulation Theory by the fact that they both stem from Cartesian dualism. In accordance with phenomenological approaches mentioned above, the theory of enaction operates on the assumption that agents’ actions can be understood “as exhibiting an inherent and ‘visible’ intentionality and as being related to each other in a meaningful way” (Fuchs and De Jaegher, 2009, 467). This is possible due to the fact that the very substance of what one means in interaction is always embodied. “Bodily expression does not mean a simple subsequent externalization of what already is inside me, but rather expression is a realization of sense” (Waldenfels, 2000, 222); “For the enactivist the body is the ultimate source of significance; embodiment means that mind is inherent in the precarious, active... process of animation… Cognition simply cannot but be embodied” (Di Paolo et al., 2010, 42). The mutual visibility of agents is explicated by the genealogy of social understanding that has a firm basis in intercorporeality. The genealogy points to basic empathy as developed in the first month of human life on the basis of perpetual corporeal interaction between the child and her most intimate caregivers (Tronick et al., 1979; Murray and Trevarthen, 1985; Kelso, 1995). This relationship is based on “trust in others and bonding capacity,” where “mismatches,” i.e., “interactive errors” are followed “with quick reparations” and reparation becomes a key process of social understanding (Fuchs and De Jaegher, 2009, 479). This leads directly to the *primacy of visibility* that is potentially able to integrate invisibility, disruption, and misunderstanding: “interactional experience continually increases the skillfulness of the participants…This is based on the ‘visibility’ of intentions-in-action” (Fuchs and De Jaegher, 2009, 471). Visibility is further reinforced by the phenomena of “coordination” as a “ubiquitous phenomenon in physical and biological systems” (De Jaegher and Di Paolo, 2007, 490) and “mutual modulation” (De Jaegher and Di Paolo, 2007, 504) that both play a crucial role in the generation of meaning called “participatory sense-making” (De Jaegher and Di Paolo, 2007). The expressive phenomenon is understood as a *unity* that is fully at stake in the process of mutual understanding.

Obviously, enactive theory does not fully deny the relevance of opacity for social interaction and understanding. Such an interpretation would be false and misleading. There are basically two strategies for dealing with the opacity or alterity of the other in social interaction. The first emphasizes the radical alterity and non-transparency of the other (e.g., Lévinas, 1979, 89, Theory Theory, Simulation Theory). However, as Zahavi (2005, 175) mentions, “the difficulty with this view is that it often tends to emphasize the transcendence and elusiveness of the other to such extent that it not only denies the existence of a functioning intersubjectivity, but also the *a priori* status of intersubjectivity.” This is indeed the reason why the enactive theory rejects such an approach. The second strategy tends to see opacity as a relevant aspect of the shared process of coping and social understanding (e.g., Husserl on interplay between ipseity and alterity: Hua 8/62; 14/457; 13/263). This perspective characterizes the participatory sense-making approach as well. It is based on the assumption of the primary visibility of the other, as I mentioned above. On this view, the other is never “totally alien” (De Jaegher and Di Paolo, 2007, 504). Because the other appears due to “my own participation in the emergence and breakdown of joint relational sense-making” (De Jaegher and Di Paolo, 2007, 504), we cannot be completely alien to each other. Authors say that agents taking part in interaction do not “experience the other-in-interaction as totally obscure and inaccessible, nor as fully transparent, but… as protean pattern with knowable and unknowable surfaces and angles” (De Jaegher and Di Paolo, 2007, 504). However, the basic unity of the shared process enables “mutual modulation” and makes visible within the process of social interaction *only those aspects* that *make sense to me* as a participant. It is a continual process where misunderstandings as the “dialectical counterpart of understanding” *serve* the initiation of the process and its continuation “like questions that lead to answers in the subsequent course of the interaction” (Fuchs and De Jaegher, 2009, 471). The assumption of the primary visibility of the other, the phenomenon of mutual modulation and coordination may be seen as characteristics of the primary readiness and tendency to understand the other, to interpret her as meaningful.

### FORMULATION OF SPECIFIC FOCUS OF THE STUDY: THE INVISIBLE EXCESS OF SENSE

Principally in agreement with this way of interpreting social cognition, I wish nevertheless to focus in some detail on one particular and rather subtle aspect of invisibility. I hope that taking this phenomenon into consideration will enable us to uncover a new dimension of opacity in social interaction besides radical hidden transcendence (the absolute form of the intangibility of the other). First of all, I wish to follow Zahavi’s (2005, 175) assumption that “the encounter with the other is, in any way, prepared and conditioned by an alterity internal to the self.” I wish to demonstrate on the basis of my experimental study that there is a “form of alterity internal to the embodied self,” that self-experience of subjectivity must contain a dimension of otherness and that intersubjectivity would otherwise be impossible (Merleau-Ponty, 1945/1962, 400–401; Zahavi, 2005, 158). Secondly, I wish to demonstrate that this otherness is not only a dialectical counterpart of our unity, not only a lack of sense that calls for fulfillment or a question that calls for an answer, but it is rather a *performative* aspect of our existence that can obtain only as invisible and can contribute to social interaction only if we let it run its course. In other words, there is an aspect of invisibility that contributes to social interaction and represents something that *makes sense* in social interaction, although it is not within the agent’s reach and comprehension. This is why I call this aspect the invisible excess of sense.

To demonstrate this sort of phenomenon and to support my thesis I wish to refer briefly to Merleau-Ponty’s description of opacity in two layers, one corporeal and one cognitive. First of all, he emphasizes that visibility is, already on the corporeal level, given under the condition of non-transparency, invisibility. Human beings are visible, because they are not transparent. In other words, one can be visible to somebody if one is invisible in some respect, if one gives resistance to perception. As Merleau-Ponty (1964b, 167) put it: “Experience is… contact of finite subject with impenetrable being… the flesh of what is perceived, this compact particle… stops exploration.” Thus, the condition of the possibility of the visibility of the other seems to be not the willingness to be visible, but rather the resistance to the view of the other. The hiddenness of perceived being is given in the density, the thickness of materiality which impedes any further seizing hold of it. Merleau-Ponty urges us not to seize hold of the real, but to become accustomed to its mystery, hiddenness – to its “being in withdrawal.” He warns that as soon as we create our possibilities, goals or ideas out of the real, we lose sight of a certain layer of things, a layer of inexhaustible riches opened to our gaze.

As regards the “cognitive” layer, Merleau-Ponty (1968, 150) speaks of ideas that can exist only in a hidden way: “It is as though the secrecy wherein they lie... were their proper mode of existence.” By this Merleau-Ponty does not mean to say that these ideas are abstract and invisible, pure, and intangible. He points out that their visibility is necessarily covered and they can exist only in a covered way: “there is no vision without the screen: the ideas we are speaking of would not be better known to us if we had no body and no sensibility; … [they] are not exhausted by their manifestations.” Merleau-Ponty thus reminds us of the complex relationship between the visible (perceptible) and invisible aspects of meaning. He states that there are “ideas” whose existence is necessarily embodied although this embodiment makes them unavoidably opaque. There is nothing like an abstract idea transformed subsequently into a sign graspable by the receiver. These ideas can exist only as hidden and never complete in their manifestation. Their being is embodied, but their meaning is not totally given by perceptible signs. It is uniquely this invisibility which gives them their performing power, their authority in the shared perceptible world. “It is that they owe their authority, their fascinating, indestructible power precisely to the fact that they are in transparency behind the sensible” (Merleau-Ponty, 1968, 150). We should understand from this that visible meaning is sometimes accompanied by the invisible aspect that amplifies the “active power” of what is being perceived. Thanks to their opacity ideas address the receivers, provoke them to action, and move them. They are performative. This view is not restricted to the thought of Merleau-Ponty. Ricouer (2000, 337), for instance, mentions the existence of the unsiginifiable element in human experience that is a source of meaning without ever being addressed.

## METHODOLOGICAL BACKGROUND

As I have already mentioned, the existence of the invisible excess of sense and the inner duality based on irreducible self-difference and self-opacity of the agent are to be shown through experimental investigation using art-based practices and methods of qualitative research.

The art-based and theater-based inspirations for the study of social interactions have a long-standing tradition in academic research. The wider context of the presented research can be seen in the work of the following researchers and their conceptions: the dramaturgical analysis of symbolic interaction in everyday life of the sociologist Goffman (1956; see also Hare and Blumberg, 1988); Berne’s (1964) transactional analysis inspired by theater practice and his view on games we all play in social interaction; sociodrama, sociometry, and psychodrama in the work of Moreno and Moreno (1975a,b, 1983) the anthropological account based on theater metaphors given by Blumenberg (1989); and the ontology of play developed by Fink (1960, 2012). If we understand individuals as agents or actors, then social interactions can be viewed as dramatic productions. The resonances and conflicts in social interaction, reflecting processes and pathologies, role play, the issue of visibility and invisibility of an individual to the other, the exposure of the actor to the public, the relationship between labor and entertainment, and inter-corporeality in social relations are studied in this context by means of thoroughly examining the agent’s experience on stage.

### “(INTER)ACTING WITH THE INNER PARTNER.” SETTING

My analysis is meant as a modest contribution to this scope of investigation. It focuses on one experiment which concerns a detailed study of self-interaction and social interaction in an empty space (stage) under special conditions. The experiment “(Inter)acting with the inner partner” is being developed by Ivan Vyskočil, Czech psychologist, pedagogue, philosopher, playwright and writer and it has been practiced at the Academy of Performing Arts in Prague for the last 20 years (Groenewald, 2004).

The practice is organized in the form of semester courses (September–January, February–June) for the general public, i.e., for people who take the course out of their own interest. Each year there are approximately 10 parallel courses of the practice. Participants are of different age, affiliation, gender, nationality, and professional background. The initial motivations of participants differ. Among them are curiosity based on the renown of the practice and author, self-knowledge and self-development, and artistic inclination. “(Inter)acting with the inner partner” is not a method focused uniquely on the training of future actors, but it provides interested people with the capacity to act openly in front of other people and to develop their consciousness of body, mind, and action. The experiment takes place in sessions. Each session lasts an hour and a half and is organized once a week for a minimum of 4 months. The significant effect of the experiment, however, is usually achieved after about a year of rehearsal. The group of participants is closed, their number being limited to 15. The session is led by one or two leaders, professionals with extensive experience in both the practice itself and taking a leadership role in it. The sessions take part in a bright and empty classroom with a high ceiling, empty space (stage), and the appropriate number of seats. The Academy of Performing Arts guarantees the ethical approval of the practice: the experiment is fully voluntary. If a participant concludes after several sessions that she does not wish to continue, she may choose to stay in the group without practicing or simply to leave. However, this case is extremely rare in spite of the fact that the practice is sometimes frustrating, especially in its initial phase. The favorable atmosphere helps people concentrate on what can be gained from the practice.

### DESCRIPTION

The experiment consists in entering the empty space (stage), to be seen by other participants, and experimenting there for a time ranging from 2 to 5 min. The experimenting participant is thus alone in a field of the onlookers’ attention without any aids (e.g., music, props, costume). She is given no task in advance, no role to play, no object to deal with. She appears in a situation of so-called “public solitude.” Public solitude is understood as a situation in which the participant does not contact the spectators in any way, especially visually or physically. It is “as if” they were not present. The spectators, however, are not fully detached observers. They are encouraged to support the actor with “favorable attention” which means that there is no loss of intersubjectivity despite the lack of discursive and direct eye contact.

The participant is encouraged within this time-frame to “go out of oneself,” “express oneself in a sufficiently intense way” and to “come back toward oneself” through voice, body expression and speech (Vyskočil, 2005, 4) ^1^. This means that the performer expresses herself in such a way that this expression attains some sort of autonomy – it becomes a meaningful “figure” (i.e., autonomous unequivocal expression) in space and time that can be addressed back to the performer and make her react as someone else.

Each individual trial is terminated after the pre-set time period by means of an auditory signal. The participant joins her seated colleagues and discusses observations and comments about what she did in the space when exactly “intense” moments with potential for acting appeared, by which condition the action could proceed, what exactly blocked the action, how the psychosomatic balance or disorder affected her capacity to be present in the situation, in front of other people, etc. The discussion is facilitated by the leader who usually gives most of the comments. After the discussion, another participant volunteers to go into the empty space and practice. There are usually two or three rounds for each participant during the session. Participants are encouraged to note and articulate their experience, discoveries, observations, and ideas in the form of regular written reflections that enable them to fix key moments in the development of the process.

### DATA GATHERING

The practice has been thoroughly developed and studied with respect to many features (self-consciousness, creativity, communication skills, psychological effects, group dynamics, etc.). The research is developed at the IRSAA (https://www.damu.cz/cs/umeni-veda-vyzkum/ustavy/ustav-pro-vyzkum-a-studium-autorskeho-herectvi) at the Academy of Performing Arts in Prague. The institute provides systematic data gathering and outcome-analysis of the mentioned practice (Vyskočil, 2005; Slavíková, 2009; Suda, 2009; Chrz, 2010a,b,c). The data gathered since 1995 up to this moment consist in:

(1) 1200 video records of rehearsals of participants and comments of prospective leaders dated from 1995 to 2014, assorted according to the individuals (enabling to study the genealogy of their development in the practice), codified and accessible in the Archive of the IRSAA, Academy of Performing Arts, Prague, Czech Republic.
(2) 1600 written reflections given by participants (hand written or digital) and leaders of the sessions – self-reflective notes of the key moments of their practice and observations, codified, assorted, and accessible in the Archive of the IRSAA, Academy of Performing Arts, Prague, Czech Republic.
(3) Interviews with Ivan Vyskočil explaining the main principles of the respective practice, some of which are available online at http://www.ivanvyskocil.cz/, or http://www.interactingwiththeinnerpartner.org/Downloads_&_Links_files/A%20Discussion%20with%20Ivan%20Vyskocil%20about%20IwIP.pdf">, codified and accessible in the Archive of IRSAA, Academy of Performing Arts, Prague, Czech Republic.

#### Data used for presented study

The results I wish to present in this paper are based on my participatory observation and dramaturgical analysis of one closed group that assembled from September 2012 to June 2013. There were in total 36 sessions, each lasting an hour and a half. The particular group consisted in 15 participants (six men, nine women), 10 of them 20–30 years old, 3 of them 30–40 years old, and 2 of them 40–55 years old. Seven participants were university students of philosophy, two IT professionals, two students of authorial acting, one unemployed, one professional translator (Czech-Japanese), one professional anthropologist, one on maternity leave. Data I used for investigating the main idea of this paper consisted in:

(1) 30 written reflections made by participants (hand written or digital) of the sessions – self-reflective notes of the key moments of their practice and observations.
(2) Field notes taken from the position of a participating observer, on the basis of interviews and collective discussions with other participants.
(3) Additional experience accumulated and recorded on the methodological basis of participatory observation made throughout my engagement for 11 years in the exercise as participant and leader.

### RESEARCH METHODS

For investigation of “(Inter)acting with the inner partner” I used the following methodology: dramaturgical analysis, participatory observation, and the phenomenological approach.

In accordance with dramaturgical analysis of Goffman, I understood participants as performers of a dramatic situation presenting themselves at the beginning of the practice “as such and such” in order to satisfy or resist the cultural norms, values and expectations (Bochner, 2001; Spry, 2001; Jago, 2002). “(Inter)acting with the inner partner” is explained by Vyskočil (1981) as a “laboratory of (inter)action in dramatic situation.” This convergence enables me to use dramaturgical analysis as a method of investigation of how identities, values, meanings, opacity, and relations are in detail constituted and executed in the stable conditions and protocols of the practice described in chap. “Description.” These features were studied with participatory observation and observation of participation (Malinowski, 1922; Firth, 1985; Tedlock, 1991, 2000; Clough, 1992). I made use of the following: direct observation as a member of the audience, participatory observation during informal meetings with other participants, in collective discussions about the practice, analyses of personal text reflections written by participants, narratives on development, and transformation of other members of the group.

For participants, the phenomenological contribution to this methodology was very important. The setting of public solitude enables them to adopt Husserl’s idea of *epoché* (for relevance of this method see, Moustakas, 1994; Sadala and Adorno, 2001; Groenewald, 2004). *Epoché* means bracketing (withdrawing from personal consideration, Groenewald, 2004, 50) the direct intentional reliance of the participant on other subjects and objects of the world. This bracketing concerns “pre-given coordination” (De Jaegher and Di Paolo, 2007, 495), obvious ways of being and thinking in public through coded, normative ways of interaction. Participants were made aware of the fact that the practice enables them to distance themselves from their automatic coded forms of performance in the public world, to become conscious of this way of acting in public and to study structures of their action without abandoning action as such (Creswell, 1998, 54 and 113; Moustakas, 1994, 90).

## RESULTS

In this section I describe results of participatory observation for 1 year and dramaturgical analysis of a group of beginners, as described in Section “Data Used for Presented Study.” As I mentioned in Introduction, my research (inspired by Merleau-Ponty’s ideas) was focused on:

(1) the exemplification of the structure of self-alterity of the agent that is constitutive for action and social interaction,
(2) the hypothesis of the existence of an invisible excess of sense in social interaction that has a performative function.

Due to the fact that the effect of the practice is not only cognitive but also transformative and develops in the course of time, the observations concerning the research at issue changed significantly with the number of sessions. For this reason I decided to divide the description of observations into stages.

### UNCANNY CHAOS

The situation of being visible for others without any task to perform and without any *a priori* given role represents the initial period of the practice. This period typically lasts between 6 and 10 sessions. It is usually described by participants as a situation of the deepest confusion, chaos, uncanny experience, frustration, embarrassment, fear, anxiety, threatening exposure, and emptiness. As personal notes of participants and interviews document this situation, the negative emotions, according to actors, stem from the fact that they cannot use their obvious codified way of social behavior. One participant writes: “When I appeared in the space, I couldnot identify myself with anything particular. I was nobody suddenly. It was unbearable.” The others add: “What is the most embarrassing is that I cannot use my usual tricks,” “There is no where to hide, I am like infinitely exposed.” The function of social roles becomes evident in the following commentaries: “If I should not have a role, I do not know what the others want from me.” “I do not feel safe if there is no role for me.” Participants agree that they do not know who they are if there is not the “pre-given coordination,” coded game of roles, the possibility of being visible “as this and this” – as a clever guy, beautiful lady, rebel, bored intellectual, engaged socialist.

The participants react to this situation during the initial experiments with a chaotic “overtension,” expressed in unlimited speaking, chaotic moving on the scene, fighting with the situation, or on the other hand, with a very remarkable “undertension,” loss of effort, depressive behavior and physical resignation, flight and freezing. Written participants’ notes document this feature again. A young participant explains: “When I first tried the IwIP I stood in the corner of the room and couldnot move, it was like I was stuck.” The reaction of another agent was different: “I only was able to run in the circles around the space faster and faster.” Participants had quite liminal reactions as well: “My first attempt looked like a very intense training in martial art,” or “I just lay down on the floor and hid my head in my arms. It was like an overwhelming ‘nothing’ all around me.”

This state can be designated with Vyskočil’s term “state of insensibility,” a sort of trance following from obvious dependence of individual on public expectations and ruled social interactions. This very frustrating initial stage, however, does not discourage participants from keeping practicing. Their curiosity is greater than the negative feelings regarding the first rehearsals. As they comment on it: “It attracts me in spite of the fact that I do not absolutely know what this can bring,” “There is something intriguing in it, I wish to find it out,” “It looks like nonsense, but I kept thinking of it the whole week. I am extremely nervous before each trial but immediately after the session I wish to try it again.”

This stage of the experiment does not show much according to our hypothesis that social interaction includes performative elements of opacity and invisibility and that this invisible excess of sense already functions on the level of self-relationship due to the essential self-opacity and self-alterity of each agent of social interaction. In terms of our hypothesis this stage of experiment is preparatory. In spite of it, it shows important aspects of visibility and invisibility in social interaction in agreement with the enaction theory:

(A) Action for a human being is always interaction. Being taken away from lively interactions, one feels uncanny and empty, powerless and “nobody.” This result validates the enaction assumption that each agent is a center of activity in the world, which means that the sense-making proceeds as enactment of the world, active exchanges with the world that are inherently significant for the cognizer (De Jaegher and Di Paolo, 2007, 487–488). In case of the lack of these lively, historically accustomed interactions with the world the precariousness of the systems appears to be significant. The isolation of the agent from the network provokes extreme reactions (fight, flight, freeze, exhaustion).
(B) The feeling of emptiness and stress when roles are taken away confirms Goffman’s assumption that agents embody dramatic roles in social interaction, that they are using stabilized normative system of communication as their way of being “somebody” in public sphere. Being visible in social interaction very often means identifying oneself with an image the agent wishes to expose in the interactions and being invisible in another way (Blumenberg, 1989, 55). Chrz (2010a, 155) points out that this level of practice uncovers the “rigid ego identified with the mask.” The social interaction proceeds then as an interplay of coded roles, habitus (Bourdieu, 1990, cit. op. De Jaegher and Di Paolo, 2007, 495).
(C) It is important to notice that participants are attracted by the practice despite the frustration it brings. The curiosity of primarily frustrated participants is a testimony to the human ability and even the will to cope with unobvious situations beyond pre-given coordination (De Jaegher and Di Paolo, 2007, 495), to wish to act otherwise than in a pre-coded way.

### PHYSICAL NON-TRANSPARENCY AS THE FIRST FORM OF INVISIBILITY

The next stage of the experiment arises after about six sessions (1 month and half of practicing). At this stage participants slowly allow themselves to calm down, to concentrate, to loosen up, to perceive and to express themselves. In this period they very often mix their tendency to imitate, copy, accept and produce various prefabrications and standards in order to amuse the observers, not to be silly in front of them, to escape from the uncanny situation etc. with a sort of acknowledgment that they are simply here as they are. Their physical presence seems to be sufficient for being visible. They become conscious of their essential non-transparency, their resistance to the gaze of others. As one participant says: “For so many weeks I have tried to perform something interesting here, but I finally found this always so stupid. So now I decided just to stand up in the center of the room. I told myself: nobody can harm me, let them watch if they want to. This is me. I was standing there for a very long time. I felt like a rock, or statue, full of meaning suddenly.”

The key observation in this stage consists in becoming *aware of oneself as of the other on the physical level.* This is documented by the following commentary: “I started touching myself with my hand and explored the boundaries of my body, those of my face, of my neck, of the other hand, of the back. It was like discovering myself in the space, physically. I realized I was there as a body. This calmed me down, it was sufficient to be there and feel my boundaries. I was there like this for the others as well.”

The awareness of self-alterity led sometimes even to creative play: “It was for the first time I really stopped focusing on what the others think of me. I was uniquely interested in the way my hand was moving around my body. It was like a small butterfly touching me at different places. And at the moment I told myself it was a butterfly, my hand was more and more like this and my body changed into a flower. It was amazing, I could just play with it.” Another form of self-alterity was found through voice communication: “I had a problem with my voice. I couldnot speak loudly, I felt ashamed. But this time I told myself: well, the worst thing that can happen is that I will be stupid before them as usual. So I cried out loud and it really scared me. It was like the voice of a stranger. But I cried back, telling it that he scared me and that he should stop immediately. And the first answered he couldnot stop until I would calm down. And I answered I couldnot calm down while he kept scaring me. And then he proposed to me that we should cry together and show we are here. It was a sudden change and even very amusing one. I completely forgot about the fear in front of the audience and laughed a lot.”

These commentaries document already some aspects present in the research hypothesis:

(A) Already on the level of physical self-affection one realizes that one relates to oneself as to another [the difference between me and myself in the sentences “I touch myself as… (a body),” “I can see myself as… (a butterfly),” “I hear myself as… (a stranger)”]. This self-alterity causes the need to respond, to act.
(B) Participants notice at this stage that their mere physical existence “makes sense” to them and to the audience without the need to produce anything. Paradoxically, they make sense because they are not transparent to themselves, because their flesh resists their own gaze and that of others. They can understand themselves always only “as” something or somebody else. They never say, “I am I,” they are not visible to themselves *as* themselves, but always as something/somebody else. They are always visible because they are invisible as a totality. They are visible because they hide themselves at the same time to the gaze of others in some respect. This is what Merleau-Ponty (1945/1962, 6) means by the concept of the indeterminate in perception. Other terms Merleau-Ponty uses with respect to this phenomenon are ambiguity, vagueness, or opaqueness. He speaks as well of the mystery of the impenetrable flesh that has performing power. I cannot see “I,” I is invisible for me as an “I.” The self-relationship contains difference and indeterminacy, opacity, non-transparency, and this very fact makes human being move and be alive. As soon as participants became aware of their natural non-transparency and thus meaningfulness they gained the power to act they had previously been missing.
(C) Participants became aware of their non-transparency for themselves together with their awareness of being non-transparent for the others. Self-interaction and social interaction seem to arise together (without one being prior to the other) from the experience of non-transparency, thus self-opacity and the possibility to engage in a non-trivial relationship.

### BACK-STAGE AND FRONT-STAGE: SOCIAL INVISIBILITY

After approximately 10 sessions, a new stage of experimentation emerges. Participants feel sufficiently assured through their physical resistance and being-in-relation with themselves through physical contact so that they start to observe the duality of roles and “non-coded behavior.” What comes to their mind very often at this stage is the idea that they are hiding something very true (“back-stage”) behind their prefabricated roles (“front-stage”). This is a very personalistic part of the experiment in which agents have the impression of uncovering the alleged “secret Self,” hidden sphere of themselves. A young participant’s description demonstrates the effect of the uncovering of secrecy that has not yet been embodied and enacted: “Up to now I have always controlled myself in order not to show the truth. But the experiments always brought me to this point. So this time I followed the impulse and transformed into a child. A child in the uterus. I had my eyes closed, was lying on the floor huddled and moved very slightly. I thought I would stay there forever because it was the most secret Self I had. But after some time, I do not know how long it took, it started to be somehow boring. It did not interest me anymore. It was very surprising for me. I stood up and told the child: it’s time to be born, don’t you think?” The way in which the utterance and exposure transform the content of a secret idea is illustrated by the following example: “When I for the first time said loudly I was stupid and again stupid and stupid, it sounded suddenly not like the only truth about myself anymore. I had to react by saying that I objected. The stupid one still insisted on being stupid and the other figure tried to tell him it was a nonsense. The secret truth transformed into a good piece of a dual game.” A more general view of the relationship between personal engagement and self-differentiation is offered by a third comment: “The more I am personal in the experiment, the more I see I have many different aspects or figures linked to each other. There is nothing like the only true Self. It is rather a dialog among different agents.”

With respect to the investigation of the mentioned hypothesis we can note at this stage of the experiment the following:

(A) Participants became aware of a new form of self-alterity in this stage of experiment. It had the form of front-stage and back-stage, the image consisting in normative roles and the secret hidden “true Self.”
(B) As the comments of participants show, once the “true Self” from back-stage is manifested, it ceases to function as the very true content of the agent. It transforms into one aspect of an interaction that calls for an answer. The enactment transforms the alleged closed mental image into an embodied form of self-interaction of the agent. This confirms again the enactivist idea of inexistence of a closed univocal mind that may be secondarily fully manifested. The sense is always made through enactment in the world and interaction (De Jaegher and Di Paolo, 2007, 488).
(C) Thus, once being enacted and embodied, the hidden Self brings along new performative possibilities hitherto unknown by the agent, the invisible excess of sense. The enacted baby gives the agent the active power to stand up on his feet. Exposed stupidity gives the agent the power to object and form another figure. Being personal turns into being multiple.

### DISCOVERY OF THE OPAQUE OTHER: SELF-RELATIONAL INVISIBILITY

The fourth stage of the experiment starts approximately after twelve sessions. With an increasing number of attempts supported by the increasing trust in the positive feedback from audience, the participants start not only to orient themselves in the situation but even to enjoy it in some respect. Enjoyment appears when the participant begins to get more relaxed, to slow down and become more curious about *what is happening* instead of focusing on oneself and one’s exposure and visibility. This transformation of focus goes from the alleged hidden self toward miniscule events that happen to the agent: for instance, a slight motion of fingers, ideas enrolling in mind, fissure in the wall, sound of steps. These events can be understood as so-called impulses, as triggers of some unknown expressive forms that are yet to be. Through the expressive amplification, the trigger develops into the so-called figure, i.e., a discernible, unequivocal, and complex expression having clear contours and meaning (for example, a slight motion of the fingers becomes slowly a mother waving goodbye to her child going to school for the first time; or a slight motion of fingers becomes a dancer in a group around a fireplace).

The following example documents this process very clearly: “I started with a slight balancing on my feet. I was balancing in this way until I realized it was like being on a ship. I balanced a little bit more and it made me wave to people who, I imagined, were waving to me. “I will get back soon” I cried out to them. “Good luck, our hero!” They cried out. (I changed into them for a while) I waved three more times and then I had a sudden impression I was completely alone on the open ocean and my waving is useless. “What shall I now do with this waving hand?” I asked. But at the same moment the hand was already acting as a magic animal who tried to bite me. My reaction was to bite back at the beast. We fought for a moment and then the signal stopped the play.” This example shows the function of imagination and playfulness at this stage of experiment. The participant is not any more concerned by himself personally. He is capable of following the “logic of the play” that has its own rules. This capacity includes the readiness to change one’s own stance, bodily scheme at the right moment of the play. The key skill in “(Inter)acting with the inner partner” is thus to follow the order of play, not that of one’s own fixed form. It includes catching the moment when the intensive expression receives “an answer from the other side” (Chrz, 2010a, 154). The other side is a name for an *a priori* unlocated answer, the emergence of a response in a situation. The other side does not mean the deeper secret Self, the alter ego, but the situational opposite, a surprising emergent phenomenon that balances the hitherto monological way of being and acting (e.g., the change from balancing to waving, from waving to crying, from crying to reflecting, from reflecting to biting).

The other example shows that the dialogical structure can appear in the discursive form as well: “I was walking in the circles in the space. After quite a long time I told myself it was too boring to walk in this way around the room. ‘Can you do something more interesting, so that I can react on it?’ I asked. ‘No’ was the response, ‘I am a very boring sophisticated philosopher who does nothing but walk in a boring way and produce boring ideas. Do you want to hear some?,’ ‘Yes please’ was the answer ‘it sounds very attractive in the end. Tell me the most boring one you have. Are you paid for this sort of thinking? How much do you get?”’ This example shows again that the agent does not identify herself with one or the other expressive figure but follows the dialog between them. She performs not as an individual agent, but as *dividual* agent, i.e., an agent capable of existing as divided, in different aspects/identities. Aspects of this alternation are not more or less essential among themselves. This playful interaction with oneself has to have specific tempo-rhythm so that it does not fade out or explode. The practitioner should respect the rules of her own play.

In the third example a participant directly points out the experience of surprise and fascination. “I walked very slowly in the space. Everything seemed to me boring. I told it aloud: ‘How the world is boring… nothing happens at all, nothing, nothing.’ But when I pronounced the word ‘nothing’ it started to interest me that it can be pronounced as a sound made by a barking dog. It was extremely surprising to me that I transformed from a bored IT into a dog, but it gave me so much energy at the same time. I was fascinated by each sound I pronounced and the situation began to clarify itself. I was so deeply immersed in the play that I even misheard the auditory signal.” This example shows that the subtle sensibility to what already happens in the situation brings new forms of meaning. The “nothing” was transformed into a source of meaning that gave the participant “energy” and “fascination.” These moments are usually very surprising because they are not *a priori* given. They accompany the standard utterances and expressions as their marginal, even invisible aspects.

The audience reaction at this stage of practice is very significant. The creative withdrawal of the participant from her personality has a paradoxical effect on observers. One observer commented as follows concerning the first example: “She is not speaking of her own accord, but yes, now she has a sparkle, something that is of her own. She is attentive and exact.” The other commented: “She fascinated me by some unknown subtlety.” The second example was commented on in the following way: “I do not know why, but his action was suddenly addressing me. It is precise and strong. I have to think on it intensively.”; “He uncovers something general in his action that attracts my attention.” The third example was commented on as follows: “There is something that influences me, fixes my attention all the time, a sort of secret that is extremely powerful.”; “I so much like the moments of subtle concentration on the play when it happens, when it starts to make sense. I seem to come alive at this moment and reflect on what is going on.”

The “subtlety” very often described by metaphors as an idiosyncratic “color,” “taste,” “sparkle” of the action is what I wish to denote as the invisible excess of sense. According to the description of participants, some ungraspable aspect of the action moves them, fascinates them, makes them attentive and reflective. The opacity of such aspects involves performative force for them. This excess could even be understood as an artistic dimension of our expressivity.

This stage of the experiment concurs with both points of the hypothesis:

(A) Self-interaction at this stage of the practice arises as dialog among different expressive aspects (figures) without being personalistic and without one being prior to the other. The creative distance from personalistic interpretation of action discovers the lively ambiguity and non-hierarchical dialog that produces meaning. To *make sense* means not to insist on some fixed identity and closure, but rather to take the situational counterpart as one’s own potential. Indeed, to make sense means to change in a consistent way. The lively identity of a person consists in the possibility of changing alongside the potential offered by the situation.
(B) The curiosity, playfulness, and relaxed sensibility amplify the probability of being able to bring into play invisible excess of meaning present in the situation. The action gains new dimension of meaning through the fact the agent is fascinated and not scared by the unknown.
(C) The audience approves of the existence of an excess of meaning, Merleau-Ponty’s “active power of what is being perceived.” Observers feel fascinated. They perceive the unobvious character of actor’s expression and of the situation. The performance attracts the observers’ attention by a certain power, moves them and makes them think at the same time.

### EXPERIMENT FOR MORE AGENTS: INTERSUBJECTIVE INVISIBILITY AND ETHICAL ATTITUDE

The final stage of the 1-year experiment consisted in interaction of more participants in the space. The experiment had the same setting as individual practice, except that now there were two participants in the space together. Their goal was to establish mutual contact using the same hints as in the individual practice (no roles, no eye contact with the audience, relaxed attention, expression). Due to the fact they all had already had experience with the practice for quite some time, their interaction had the character of relaxed and attentive improvisation that took into consideration the opacity of another human being in the space. As the following comment shows, participants were able to “give space” to each other: “The appearance of my colleague brought into play a new form of opacity. I did not know exactly what he meant and wanted, but I knew it was necessary to wait for a while. I made some movements with my hands to indicate a widening of space. Then he started to sing.” However, they agree as well that their inner dialogical structure resembles that of interaction: “For me interacting with another person was not that different from the individual rehearsal. She was there as I am for me, as another. I interacted with her as with another inner partner. She surprised me, which created a good field of energy.”

We can see that participants played along with the opacity of the other without the need to address it directly. This stance includes the acceptance that there are aspects in social interaction that are not any of our business, even though they participate in the situation. This moment raises questions on the ethical attitude toward the invisibility of the other, or the ethical extent of intersubjective invisibility.

### EXPERIMENT AND REAL SOCIAL INTERACTION

“(Inter)acting with the inner partner” brings people in an experimental situation that is lacking in relative obviousness. In spite of this, the experiment has direct implications for everyday intersubjectivity.

(1) By means of the method of phenomenological bracketing of what is common, one suppresses obvious and habitual patterns in favor of the less obvious but not less important aspect of creative interaction. This aspect is present in everyday intersubjectivity in the moments of surprise, improvisation, loss of fluency, when the agent is able to work with these moments creatively and not in a rigid (even neurotic) way. The practice gives space to investigate the very structure of playfulness in social interaction.
(2) The situation of public solitude amplifies again a marginal, but very natural state of self-interaction in solitude. Interacting with oneself is not an artificial practice, it is a genuine part of self-relationship. The practice shows how the patterns of self-interaction co-arise with patterns of social interaction.
(3) The practice is not only uncovering certain patterns, but it is transformative as well in terms of emphasizing these patterns in social skills of the participants. Participants developed during the practice joyful, relaxing, and generous ways of social interaction. They achieved the capacity of consciously stepping out of rigid habits and contributing to participatory sense-making through their specific relaxed concentration. This concentration enables development and performance of surprising, invisible but profound aspects of the other. Comments collected through interviews demonstrate it: “‘(Inter)acting with the inner partner’ changed completely my view of my surroundings. I observe people differently, try to develop our interaction from other sources, not from the norms and this sort of stuff”; “When I take the metro, I observe how people interact when they have little space or when they are in a hurry. It is very funny to see it as a game. And it is even funnier if I propose some new way of behavior there, as I once made something like sport commentary about who will be the first to step into the wagon and people immediately relaxed and started to laugh”; “Every social situation can be creative. I feel like a part of a vast network where surprising things may happen, like gifts from nowhere. They are to be noticed merely.”

## CONCLUSION

I hope to have demonstrated by means of an empirical exemplification the following conclusion: social interaction includes elements of opacity and invisibility. These elements play a particular role in social interaction. This role is performative. This means that opacity is neither an obstacle to be overcome by means of a more precise understanding, nor a lack of meaning, but an excess of meaning, a “hiddenness” of the real that has an “active power” (Merleau-Ponty). The description of the practice showed that we can sensitize ourselves to the invisible excess of sense on the physical level, on the level of social norms, and on those of self-relationship and intersubjective relationships. Aspects of invisibility are partially described by the enaction theory and in phenomenology. My goal was to underline mainly two important aspects of invisibility that have not yet been developed in detail in participatory sense-making theory.

The first point concerns the self-opacity of each agent of social interaction and the dividual, dialogical character of her self-interaction. The self-relationship is characterized as the ability to see oneself as another. It occurs as an interaction among non-identical aspects that correspond together on the basis of a temporal rhythm and the regularity of their dynamics. This observation raises new questions for further research, especially concerning the autonomy of an agent in social understanding. The theory of participatory sense-making proposes an idea of “multi-dimensional complex of identities that co-exist in what we call a subject” (De Jaegher and Di Paolo, 2007, 503), but this element has not been developed in detail in terms of its unity. Instead the autonomy of living systems is defined as “the property of operational closure” and “the virtue of their self-generated identity as distinct entities” (De Jaegher and Di Paolo, 2007, 487). Can this definition of unity be explained or even shifted toward the self-interaction-based definition that includes non-hierarchical multiplicity of different aspects? May the agent be coherent on the basis of rhythm of some inner “process,” even “play,” “dialogical order,” “dividual dynamics” (as opposed to individuality) and exactly as such take part in social interaction? How is the detachment from oneself (inner non-identity) important for human autonomy?

The second point concerns the invisible excess of sense in social interaction that represents neither a closed content of disembodied mind nor a clearly embodied expression but still has to be accepted as source of meaning. The resulting issue that arises from the idea of existence of invisible excess of sense concerns the form of the enactive approach to this sort of phenomena. How do people approach in an appropriate way the opacity of others? Does the only way consist in understanding the other, in the effort to catch what the other means, in coordination and coupling? Should a hint of hidden excess present a trigger for an attempt to keep asking, to uncover it in interaction, to understand it better through action? Does the invisibility have only a form of question, lack of sense that calls for an answer? Can we notice a certain dimension of silence, peace, pause, shutdown of dynamics with respect to the element of invisible excess of sense in social understanding? Enactive ethics (Colombetti and Torrance, 2009) is usually characterized by the focus on interactive and interpersonal dimensions of moral phenomena. This approach allows us in a most appropriate way to avoid ethical individualism. Within this very propitious ethical context I wish however, to stress that social understanding may also imply a capacity of generous respect for alterity – stepping back, letting be – which is not passive but creative.

## Conflict of Interest Statement

The author declares that the research was conducted in the absence of any commercial or financial relationships that could be construed as a potential conflict of interest.

## Footnotes

1 In order to emphasize and support this capacity, the leader of the session asks the participants at the outset to bring to mind, for the sake of guidance, the experience of being with oneself in interaction, talking to oneself, playing by oneself in pure solitude. It usually happens when we are alone and either face an issue (as we make a decision and hesitate, as we remember an embarrassing situation) or we are bored or relaxed (making faces in the mirror during a long elevator ride, singing, and narrating in the bathroom). Such basic living situations already show a certain non-trivial interactive self-relationship of human being in moments of emptiness, lacking any explicit interaction with other objects or subjects. The question is: what happens if this playful interaction with oneself as another is induced in front of the others?
